# Bullying victimization and suicidal ideation among Chinese adolescents: a moderated mediation model of depressive symptoms and perceived family economic strain

**DOI:** 10.1186/s12889-025-21579-w

**Published:** 2025-01-30

**Authors:** Xiaoyu Jiang, Qiaoyue Wei, Wenwen Yin, Shuibo Pan, Chenyangzi Dai, Linhua Zhou, Chunyan Wang, Xiujin Lin, Junduan Wu

**Affiliations:** 1 The Second Affiliated Hospital of Guangxi Medical University, Nanning, 530007 China; 2 School of Public Health, Wuhan University, Wuhan, 430071 China; 3 Science and technology college of Hubei university of arts and science, Xiangyang, 441025 China; 4 School of Public Health, Guangxi Medical University, Nanning, 530021 China; 5 School of Clinical Medicine, Guangxi Heath Science college, Nanning, 530023 Guangxi China; 6 Research Center of Adolescent Psychology and Behavior, School of Education, Guangzhou University, Guangzhou, 510006 China

**Keywords:** Adolescent, Suicidal ideation, Bullying victimization, Depressive symptoms, Economic strain

## Abstract

**Background:**

There is substantial evidence linking bullying victimization to suicidal ideation, but the mechanism behind this link is not well understood. This study investigates whether depressive symptoms mediate the relationship between bullying victimization and suicidal ideation, and whether perceived family economic strain moderates this mediation.

**Methods:**

Data were collected from 7,702 adolescents (mean age = 14.74 ± 1.69 years, 52% girls) using a self-report questionnaire that assessed bullying victimization, depressive symptoms, suicidal ideation and perceived family economic strain. Mediation and moderated mediation analyses were conducted using the PROCESS macro in SPSS.

**Results:**

Suicidal ideation was significantly associated with both bullying victimization and depressive symptoms, as determined by linear regression analysis (both *p* < 0.001). The relationship between bullying victimization and suicidal ideation was partially mediated by depressive symptoms, accounting for 66.74% of the effect. Additionally, perceived family economic strain could moderate the link between bullying victimization and depressive symptoms (β=-0.017, *p* < 0.001), indicating that increased perceived family economic strain attenuates the impact of bullying victimization on depressive symptoms.

**Conclusion:**

Our research affirms bullying victimization substantial influence on adolescent suicidal ideation, with depressive symptoms mediating this link. The study also reveals that perceived family economic strain moderates this relationship, indicating the necessity for interventions that address both psychological and economic factors to holistically support the mental health of bullied adolescents.

**Supplementary Information:**

The online version contains supplementary material available at 10.1186/s12889-025-21579-w.

## Introduction

Suicide results in the loss of over 700,000 lives worldwide each year, ranking as the third leading cause of death among individuals aged 15 to 19 [1]. It is a complex phenomenon that unfolds in three stages: suicidal ideation, suicide attempts, and completed suicide, progressing from contemplation to actual action. Suicidal ideation, which includes thoughts, considerations, or preparations for suicide, has consistently been identified as a strong predictor of actual suicidal behavior [2]. Recent research, including a meta-analysis of 103,309 adolescents, revealed that 15.4% of Chinese adolescents experience suicidal ideation [3], surpassing the global prevalence of 14% reported by Biswas [4]. Understanding suicidal ideation as a multifaceted issue necessitates an exploration of the factors that specifically affect Chinese adolescents. Drawing on Bronfenbrenner and Morris’s ecosystem theory [5], we recognize that individual development is intricately influenced by multiple environmental factors. Notably, the microsystem, especially peer influence, and the mesosystem, linking various microsystems like family and school, significantly affect development. During adolescence, peer interactions are pivotal; positive ones promote healthy growth, while negative encounters like bullying can lead to mental health issues like depression [6]. To fully understand Chinese adolescents today, we must consider the multilevel impacts of the exosystem (indirect influences such as family dynamics), macrosystem (economic factors), and chronosystem (temporal changes). The exact underlying mechanisms are still not fully known, despite the fact that numerous studies have demonstrated a connection between adolescent bullying victimization and suicidal ideation [7, 8]. The purpose of this study is to examine the association between bullying victimization and suicide ideation in Chinese adolescents, with a focus on the mediating role of depressive symptoms and the moderating effect of perceived family economic strain.

## Bullying victimization and suicidal ideation

According to Olweus, bullying victimization encompasses physical aggression (hitting, shoving), verbal abuse (name-calling), and relational aggression (social exclusion, rejection) [9]. This form of victimization intentionally inflicts enduring harm. In China, adolescent bullying is on the rise, with national research indicating a prevalence of 10.89% [10], Shandong reporting 11.59% [11], and a meta-analysis placing the overall prevalence of bullying victimization at 22.7% [12]. Empirical research and a thorough meta-analysis have consistently linked bullying victimization to mental health issues and behaviors, including suicidal ideation [13–17]. Studies across countries, such as those in Argentina, Panama, and Saint Vincent and the Grenadines, show that adolescents experiencing cyberbullying have significantly higher odds of suicidal ideation compared to those who do not [18]. A national survey of 14,603 adolescents aged 14–18 found, using binary logistic regression, that those experiencing both school bullying and cyberbullying had 3.26 times the odds of suicidal ideation [7]. Longitudinal studies have demonstrated a relationship between bullying victimization and subsequent suicidal ideation [19]. For instance, the Quebec Longitudinal Study of Child Development found that peer victimization at age 13 predicted suicidal ideation two years later (OR = 2.27) [20]. The detrimental effects of bullying victimization extend beyond adolescence, contributing to ongoing mental health challenges in adulthood [6, 21]. Escape Theory posits that adolescents confronted with bullying, particularly when lacking effective coping mechanisms, such as adequate financial support [22], may develop an intensified desire to escape their circumstances. This compulsion can drive some victims to contemplate suicide as a perceived ultimate solution to their overwhelming distress and a means to achieve the escape they desperately seek [23].

### The mediating role of depressive symptoms

Depressive symptoms, characterized by persistent low mood, anhedonia, and diminished interest, have been recognized as critical elements linking bullying victimization to suicidal ideation [24]. In the context of Chinese adolescents, the prevalence of depressive symptoms is significantly elevated at 34.24% [25], surpassing the global average of 25.2% [26]. Findings from meta-analyses reveal that adolescents exhibiting depressive symptoms are at an increased risk of engaging in suicidal behavior [27]. Furthermore, research consistently indicates that the presence of depressive symptoms heightens the likelihood of adolescents experiencing suicidal ideation [28–30]. Based on this evidence, we assert that depressive symptoms serve as a substantial correlation factor of suicidal ideation among adolescents.

Existing researches have demonstrated a link between bullying victimization and a higher likelihood of depressive symptoms in adolescents. Traumatic experiences, including adverse childhood experiences and bullying victimization, have been identified as key factors that can exacerbate an individual’s susceptibility to depressive symptoms [31–33]. Meta-analysis results indicate that bullied children and adolescents have a 2.77 times higher risk of depression compared to non-bullied peers [34]. Furthermore, longitudinal research consistently shows that bullying victimization has a lasting impact on depressive symptoms. Specifically, more frequent incidents of peer victimization in fifth grade are associated with more severe depressed symptoms in seventh grade [35]. Being a victim of both traditional and cyberbullying at the age of 15 is a strong predictor of depressive symptoms two and five years later [36]. Moreover, further research has proven that depressive symptoms mediate the relationship between bullying victimization and suicidal ideation [37, 38]. Given the interconnectedness of depressive symptoms, bullying victimization, and suicidal ideation, we hypothesize that depressive symptoms may function as a mediator in the relationship between bullying victimization and suicidal ideation.

### The moderating role of perceived family economic strain

In China, the cultural imperative of ‘raising children to be dragons’ places immense economic and social importance on higher education, creating both motivation and psychological stress, especially for economically disadvantaged families [39–41]. Empirical data reveal that low-income households allocate a staggering 56.8% of their income to education, a figure substantially higher than the 10.6% spent by high-income families [42]. This economic pressure is exacerbated for certain ethnic minorities residing in harsh environments and facing additional development barriers. For these families, the struggle to provide educational opportunities for their children is particularly acute [43]. Perceived family economic strain, the subjective sense of pressure brought on by insufficient funds to meet basic needs, has been linked in numerous studies to adolescents’ psychological well-being and adjustment [44–47]. The social causality hypothesis suggests that socioeconomic hardship, including financial stress and reduced social capital, increases the risk of mental illness [48]. Prior research has shown a negative correlation between financial stress and suicidal ideation, depressive symptoms, and bullying victimization [49–51].

Additionally, several studies have explored the intricate interplay between risk factors and financial stress in their impact on mental health. Specifically, individuals who are victims of bullying often lack the financial means to alleviate their psychological distress, resulting in a combined stress from bullying victimization and severe financial troubles within the family that substantially elevates the risk of developing common mental health issues [52]. The deterioration of social relationships, exemplified by bullying victimization, has long been identified as a significant risk factor for suicidal and self-injurious behaviors. When compounded by economic stress, these factors exhibit a synergistic effect, significantly amplifying the risk of suicide [53]. Furthermore, when individuals exhibit depressive symptoms and face financial obstacles in accessing antidepressant treatment, this prolongs the delay in receiving treatment and exacerbates the severity of consequences [54].

In summary, given the detrimental impacts associated with perceived family economic stress, this heightened level of stress emerges as a pivotal risk factor for bullying victimization, depressive symptoms, and suicidal ideation, ultimately contributing to adverse mental health outcomes. Moreover, its interaction with other related risk variables exacerbates these poor mental health outcomes. While prior research has examined the moderating effect of perceived economic strain on caregivers’ and children’s BMI and paternal depression-induced hostility [55, 56], the moderating influence of perceived family economic strain in models connecting bullying victimization, depressive symptoms, and suicidal ideation remains unconfirmed. Therefore, to better understand its critical role in the field of adolescent mental health, especially among adolescents from diverse ethnic backgrounds, this study aims to ascertain whether perceived family economic strain can function as a moderator in these relationships.

## Current study

**Fig. 1:**
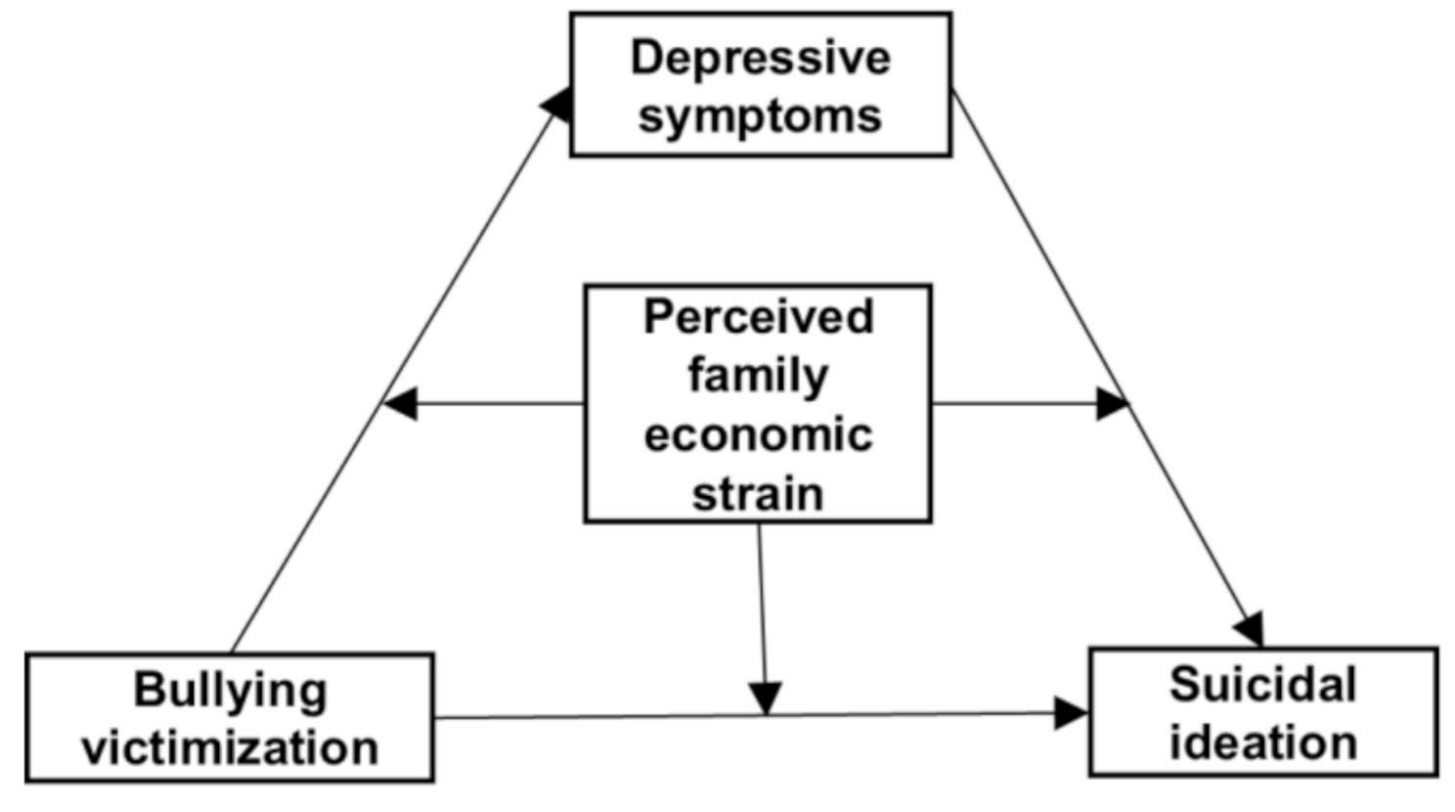
Schematic model of perceived family economic strain as a moderator of the mediation model (Model 59)

Previous studies on bullying victimization and suicidal ideation have been conducted, but limited attention has been given to the role of perceived family economic strain. This study aims to address this gap by exploring the mediating effect of depressive symptoms and the moderating effect of perceived family economic strain in this relationship. It is hypothesized that bullying victimization may positively associate with suicidal ideation in adolescents (hypothesis 1), with depressive symptoms mediating this relationship (hypothesis 2). Additionally, perceived family economic strain is expected to moderate the effect of bullying victimization, depressive symptoms and suicidal ideation. More specifically, the negative effect of bullying victimization on suicidal ideation would be greater among those with a higher level of perceived family economic strain (hypothesis 3). The comprehensive conceptual model of this study is illustrated in Fig. 1.

## Methods

### Participants

A cross-sectional study in Guangxi Zhuang Autonomous Region from November to December 2022 utilized a multi-stage cluster random sampling method. In stage 1, two cities were selected respectively from the high and low grades (and one from the intermediate grade) based on Guangxi’s GDP ranking, for a total of five areas chosen. With assistance from the local educational office, four to five junior and senior high school per city were selected for stage 2. In stage 3, school administrators assisted in the random selection of two classes from each grade (7–12) in each school. With written informed agreement from students and guardians, a self-report questionnaire was distributed to all students in the chosen courses. The survey was conducted voluntarily and anonymously, and all information was kept strictly confidential. Guangxi Medical University’s Ethics Committee gave its approval to the project (Approval number: 20200016). The Declaration of Helsinki was followed when conducting the study.

After excluding 434 students due to illogical or missing data, the final analysis included questionnaires from 7702 students (3699 boys (48%) and 4003 girls (52%); average age 14.74 ± 1.69 years). The actual response rate was 94.67% [(7702/8136) *100]. The participant characteristics are presented in Table 1.

**Table 1:** The characteristics of adolescent (*n* = 7702)

Variable	Mean (SD)/*n*(%)
Age(years), Mean (SD)	14.74(1.69)
12-14.9	4230(54.9)
15–18	3472(45.1)
Gender	
boy	3699(48)
girl	4003(52)
Place of residence	
city	3598(46.7)
town	988(12.8)
rural	3116(40.5)
Ethnicity	
Han	4037(52.4)
minorities	3665(47.6)
Only child	
yes	1168(15.2)
no	6534(84.8)
Caregiver(s)	
parent	5730(74.4)
grandparent	1773(23)
relatives	199(2.6)

Note: SD, Standard Deviation

### Measures

#### Bullying victimization

The Personal Experiences Checklist Short Form (PECK-SF) is a 14-item self-report measure designed as a condensed version of the PECK, tailored to evaluate past experiences of bullying victimization [57]. The original PECK was developed by Hunt [51], and the PECK-SF is a shortened version of it. The PECK-SF measures four distinct types of bullying victimization: verbal-relational, physical, cyber, and cultural, and provides a cumulative total score. Each item is rated on a 4-point scale ranging from 1 (never) to 4 (always). Total scores range from a minimum of 14 to a maximum of 56. The Chinese adaptation of the PECK-SF has proven to be reliable and valid among Chinese adolescents [58]. The confirmatory factor analysis results for the PECK-SF indicated that χ^2^/df was notably influenced by the sample size, yet the remaining fit indices were found to be satisfactory (χ^2^/df = 45.653, RMSEA = 0.066, RMR = 0.009, GFI = 0.952, AGFI = 0.929, CFI = 0.941). The Cronbach’s α coefficient in this study was 0.878. Overall, these findings confirm that the PECK-SF is well-suited for assessing experiences of bullying victimization among Chinese adolescents.

#### Depressive symptoms

The Center of Epidemiological Studies Depression Scale for Children (CES-DC) was developed by Radloff to assess depression-related symptoms among adolescents and later translated and adapted by William Li for use in China [59, 60]. This 20-item scale evaluates depression-related symptoms experienced in the past week, with responses scored on a four-point Likert scale ranging from 0 (not at all) to 3 (most days). The total score ranges from 0 to 60, with higher scores indicating more severe depressive symptoms, and a commonly used cut-off score of 16 or above suggests a propensity for depressive symptoms. Previous studies have validated the scale’s high suitability for Chinese adolescents, showing its efficacy in measuring the intended constructs within this population [61, 62]. The CES-DC exhibited excellent internal consistency and reliability among the sample, with a Cronbach’s α coefficient of 0.942.

#### Suicidal ideation

The Positive and Negative Suicide Ideation (PANSI) Inventory, developed by Osman to assess positive and negative suicidal ideation across two dimensions among adolescents [63], and later translated and adapted by Chen for use in China [64]. The 14-item scale assesses suicidal ideation in adolescents, with responses rated on a 5-point Likert scale, ranging from 0 (none at all) to 3 (most of the time). The overall score ranges from 14 to 70, with higher scores indicating more severe suicidal ideation. Prior research has confirmed the inventory’s high suitability for Chinese adolescents, demonstrating its efficacy in measuring the relevant constructs within this demographic [65–67]. In our study, the Cronbach’s α coefficient for the PANSI was 0.879.

#### Perceived family economic strain

The Perceived Family Economic Strain scale is utilized to assess the perceived economic strain within families of Chinese school students [68], which is the Chinese version of the Wadsworth’s Economic Stress Scale [46]. This scale comprises four items related to different aspects: food, clothes, housing, and transportation. These items include statements such as “We didn’t have enough money for new clothes,” “My parents didn’t have enough money for the food I like to eat,” “We can’t afford a nice house,” and “There’s no money left to do something fun as a family.” Participants were required to indicate the frequency with which their families encountered economic strain over the past year using a 5-point Likert scale ranging from 1 (never) to 5 (always). Scores on the scale ranged from 4 to 20, with higher scores reflecting greater levels of perceived family economic strain. It has been confirmed by previous studies to be highly suitable for Chinese adolescents, effectively capturing the relevant constructs within this demographic [69]. In our study, the Cronbach’s α coefficient for the scale was 0.844.

#### Covariates

Potential covariates encompass adolescents’ self-reported age (derived from their date of birth), gender (1 = male, 2 = female), whether they are only children (1 = yes, 2 = no), and caregiver (1 = parent, 2 = grandparents, 3 = other relatives). The models are adjusted based on these specified covariates.

### Statistical analysis

In the first step of the analysis, the continuous variables were represented as mean (Standard Deviation, SD), while the categorical variables were depicted as proportion [n (%)]. Subsequently, the correlations among the variables, including bullying victimization, depressive symptoms, suicidal ideation, and perceived family economic strain, were computed in SPSS 26.0. Moving on to the second step, the mediation and moderated mediation models were examined using the PROCESS macro for SPSS, as suggested by Hayes [70]. Initially, model 4 was employed to investigate whether the link between bullying victimization and suicidal ideation was mediated by depressive symptoms. The significance of the mediation was determined based on whether the 95% confidence interval (CI) of the indirect effect did not include 0. Following this, model 59 was utilized to explore the moderated mediation effect, examining whether perceived family economic strain had a moderating impact on the indirect and direct effects of the mediation. A significant moderated mediation effect was identified when the 95% confidence of the interaction not include 0. The bias-corrected 95% confidence interval (CI) was established through 5000 bootstrapped resamples. Furthermore, model 59 was evaluated for samples of ethnicity (Han and minority) and place of residence (rural, town, and city), respectively, to further bolster the results’ robustness. Control variables such as age, gender, only child, and caregiver were incorporated, while standardization was applied to the study variables to ensure consistency in the analysis process.

## Results

### Common method bias

To control the potential common method deviations stemming from self-report questionnaires, measures were organized individually, and techniques such as anonymous survey and reverse scoring in certain items were employed. Harman’s single factor analysis was conducted to examine common method deviations [71]. The analysis revealed that the first factor accounted for 27.3% of the total variance, falling below the critical threshold of 40%. This result indicates that there was no significant common method bias present in the study.

### Correlations among bullying victimization, depressive symptoms, perceived family economic strain, and suicidal ideation

Table 2 displayed the Pearson correlations along with the means and standard deviations of the variables. The findings revealed that bullying victimization showed positive associations with suicidal ideation (*r* = 0.355, *p* < 0.001), depressive symptoms *(r* = 0.476, *p* < 0.001), and perceived family economic strain *(r* = 0.262, *p* < 0.001). Additionally, suicidal ideation exhibited a positive correlation with depressive symptoms (*r* = 0.652, *p* < 0.001) and perceived family economic strain *(r* = 0.208, *p* < 0.001). Moreover, perceived family economic strain was positive associations with depressive symptoms *(r* = 0.240, *p* < 0.001).

**Table 2:** The correlation analysis

	1	2	3	4
1. Bullying victimization	1			
2. Depressive symptoms	0.476***	1		
3.Suicidal ideation	0.355***	0.652***	1	
4. Perceived family economic strain	0.262***	0.240***	0.208***	1
M	17.64	13.06	26.34	7.09
SD	4.39	10.00	8.63	3.48

Note: M, Mean, SD, Standard Deviation; ***: *p* < 0.001

### Linear regression analysis

The linear regression results were showed in Table 3. We found that bullying victimization was significantly positively associated with suicidal ideation in Model 1 (*β* = 0.378, *p* < 0.001) and significantly positively associated with depressive symptoms in Model 2 (*β* = 0.472, *p* < 0.001). Furthermore, in Model 3, both bullying victimization and depressive symptoms were positively associated with suicidal ideation (*β* = 0.076 and 0.639, respectively, both *p* < 0.001). Age, Gender, Only child, Caregiver(s) are analyzed as control variables in Models 1 to 3.

**Table 3:** Linear regression analysis in the paths of the mediation model for suicidal ideation

Variables	Model 1(SI)			Model 2(DS)			Model 3(SI)		
	β	Std.B	t	β	Std.B	t	β	Std.B	t
BV	0.743***	0.378***	35.771	1.075***	0.472***	47.223	0.15***	0.076***	8
DS							0.551***	0.639***	66.501
Gender	1.943***	0.112***	10.770	2.019***	0.101***	10.216	0.829***	0.048***	5.731
Age	0.094	0.018	1.747	0.588***	0.099***	10.013	-0.23***	-0.045***	-5. 364
Place of residence	0.127	0.014	1.22	-0. 144	-0.013	-1.255	0.207*	0.022*	2.483
Ethnicity	-0.032	-0.006	-0.54	-0.098	-0.015	-1. 495	0.022	0.004	0.372
Only child	-0.206	-0.009	-0.809	-0. 511	-0.018	-1.83	0.076	0.003	0.322
Caregiver	0.824***	0.048***	4.483	0.909***	0.046***	4. 517	0.322*	0.019*	2.198
R^2^	0.169			0.257			0.473		
F	224.131			379.675			861.603		

Note: *β*: unstandardized regression coefficient. *Std.B*, standardized regression coefficient. *: *p* < 0.05; ***: *p* < 0.001. Age, Gender, Only child, Caregiver(s) are analyzed as control variables in Models 1 to 3. BV: Bullying victimization; SI: Suicidal ideation; DS: Depressive symptoms

### Mediation analyses

**Fig. 2:**
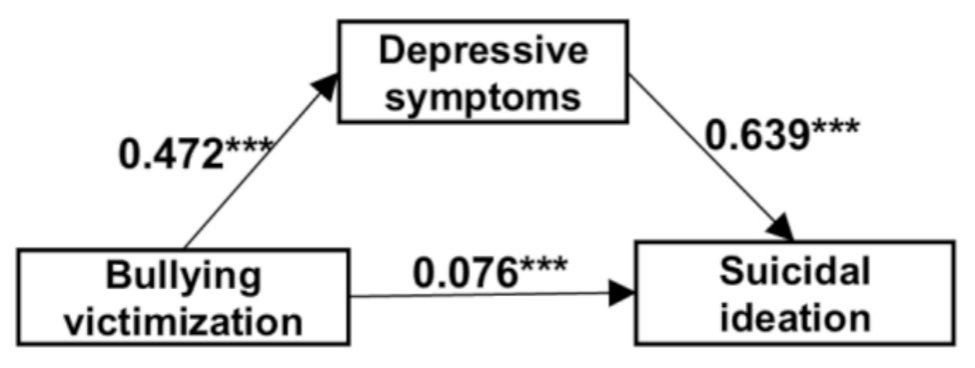
Mediation model. ***: *P*<0.001

Significant correlations existed among bullying victimization, depressive symptoms and suicidal ideation for adolescents. Additionally, bullying victimization and depressive symptoms could be used to predict suicidal ideation. Thus, the mediation analysis was used to further test the relationship among these variables. The mediation analysis results are shown in Table 4; Fig. 2. Depressive symptoms mediated the relationship between bullying victimization and suicidal ideation, accounting for 66.74% of the total effect (the 95% *CI* of bootstrapping did not include 0).

**Table 4:** Results of the mediation model on the relationship between bullying victimization and suicidal ideation

Model path	Effect	95%CI	Proportion of total effect (%)
Total effect	0.451		
Direct effect (BV→ SI)	0.150	(0.114, 0.187)	33.26
Total indirect effect (BV →DS →SI)	0.301	(0.284, 0.320)	66.74

Notes: 95%*CI*: the 95%confidence interval; Age, Gender, Only child, Caregiver(s) are analyzed as control variables. BV: Bullying victimization; SI: Suicidal ideation; DS: Depressive symptoms

### Moderated mediation analyses

**Fig. 3:**
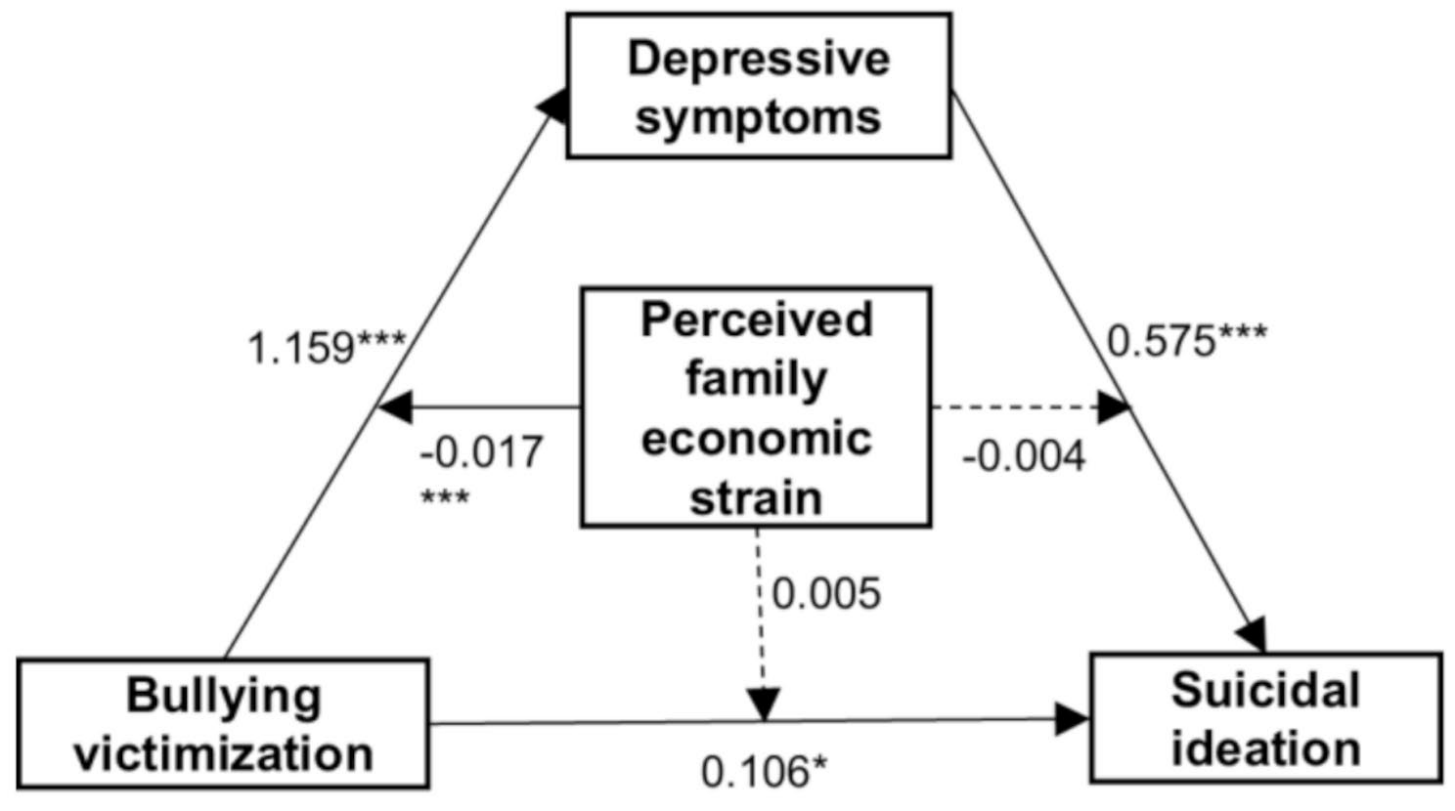
Moderated Mediation model. ***: *P*<0.001

**Fig. 4:**
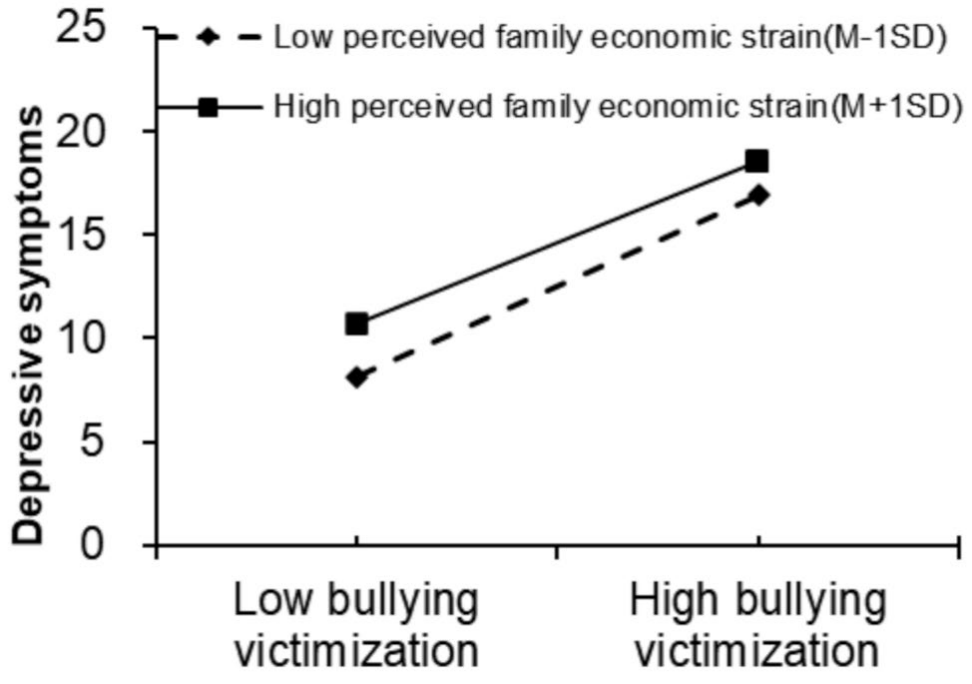
Moderating effect of perceived family economic strain on the indirect effects of bullying victimization on depressive symptoms

Moderated mediation analyses were conducted to investigate the potential moderating effect of perceived family economic strain on the relationship between bullying victimization and suicidal ideation. We employed Model 59 within the PROCESS framework, incorporating total scores of perceived family economic strain. The detailed outcomes of the moderated mediation analysis are presented in Table 5 and visually illustrated in Fig. 3. Specifically, the result revealed a statistically significant interaction effect between bullying victimization and perceived family economic strain on depressive symptoms [*β*=-0.017, 95% *CI*= (-0.028, -0.007)], indicating that perceived family economic strain moderates this relationship. However, no statistically significant interaction was observed between bullying victimization and perceived family economic strain on suicidal ideation [*β* = 0.005, 95% *CI*= (-0.003, 0.014)], nor between depressive symptoms and family economic strain on suicidal ideation [*β*=-0.004, 95% *CI*= (-0.008, 0.001)]. Further simple slope analysis demonstrated that both low and high levels of perceived family economic strain significantly moderated the relationship between bullying victimization and depressive symptoms. Specifically, the positive correlation effect of bullying victimization on depressive symptoms decreased from *β* = 1.09 to *β* = 0.976(Table 6). These findings suggest that as perceived family economic strain increases, the impact of bullying victimization on the risk of depressive symptoms diminishes (see Fig. 4).

Subgroup analysis was carried out for several regions (rural, town, and city) and ethnic groupings (Han and ethnic minorities) in order to further guarantee the results’ robustness. The findings indicated that, among the five subgroups, only ethnic minorities’ perceived family economic strain moderated the relationship between bullying victimization and depressive symptoms [*β*=-0.022, 95% *CI*= (-0.037, -0.006)]; the other two routes in the model were unaffected ((Supplementary Table 1)). Specifically, the positive correlation effect of bullying victimization on depressive symptoms decreased from *β* = 1.076 to *β* = 0.926 ((Supplementary Table 3)). However, perceived family economic strain did not act as a moderating factor in the mediation model among Han, rural, town, and city adolescents. In the supplementary material we provide more information on the results of the subgroups.

**Table 5:** Moderated mediation analysis

Variable	Model 1(DS)			Model 2(SI)		
	β	t	95%CI	β	t	95%CI
BV	1.159***	23.477	(1.062, 1.255)	0.106*	2.569	(0.025,0.186)
PFES	0.626***	6.281	(0.430, 0.821)	0.074	0.343	(-0.119,0.170)
DS				0.575***	31.891	(0.540, 0.610)
BV*PFES	-0.017***	-3. 309	(-0.028, -0.007)	0.005	1.194	(-0.003, 0.014)
DS*PFES				-0.004	-1. 702	(-0.008, 0.001)

Note: 95%*CI*:the 95%confidence interval. *: *p* < 0.05; ***: *p* < 0.001. Age, Gender, Only child, Caregiver(s) are analyzed as control variables. SI: Suicidal ideation; BV: Bullying victimization; PFES: Perceived family economic strain; DS: Depressive symptoms

**Table 6:** The moderating effect of high and low perceived family economic stress levels on bullying victimization and depressive symptoms

Perceived family economic strain		Effect	t	95%CI
M-1SD	4	1.09	33.657	(1.026, 1.153)
M	7.093	1.036	43.297	(0.989, 1.083)
M + 1SD	10.577	0.976	37.942	(0.926, 1.026)

Note: 95%*CI*:the 95%confidence interval

## Discussion

In the midst of the escalating issue of bullying victimization among Chinese adolescents, this study sought to investigate how bullying victimization impacts suicidal ideation among this population. While the significant influence of bullying victimization on suicidal ideation has been broadly acknowledged [72, 73], the precise roles of perceived family economic strain in this context within China’s evolving economic and cultural landscape remained uncertain. This research sought to clarify how family economic strain influences this relationship, explore ethnic differences in this effect, and analyze depressive symptoms’ potential mediation, as well as perceived family economic strain’s moderation of this process.

### Bullying victimization and suicidal ideation

Bullying victimization is strongly linked to suicidal ideation among adolescents, as supported by prior research and this study’s findings [7, 74, 75]. Bullying victimization often suffer from negative impacts on their physical and mental health, including anxiety, depression, and low self-esteem, which can diminish their sense of belonging and self-worth. According to the Interpersonal Suicide Theory (ITS) [76], suicide is more likely when individuals have both the desire and the ability to act on it, which includes a frustrated sense of belonging and a perceived burden. Bullying can exacerbate these feelings, leading to social alienation and, in vulnerable individuals, suicidal ideation. Therefore, preventing bullying is crucial for reducing the risk of suicidal ideation in adolescents.

### The mediating role of depressive symptoms

In this study, we tested the second hypothesis that depressive symptoms serve as a mediator in the relationship between bullying victimization and suicidal ideation, aligning with previous research [37, 77]. According to the General Stress Theory, adolescents who endure bullying or maintain poor peer relationships may experience heightened stress [78]. This stress can initiate a series of adverse emotional responses, such as fear, anxiety, anger, and sadness, which may subsequently lead to maladaptive behaviors or thought patterns [79]. When considered alongside ITS [76], bullying victimization may cause adolescents to negatively evaluate themselves and their environment, leading to internalizing problems like depression. Under depression, adolescents may be more prone to feelings of being overwhelmed and a loss of belonging, fulfilling the initial components of the ITS and paving the way for suicidal ideation. Additionally, research indicates that individuals exposed to bullying may experience dysfunction in the hypothalamic-pituitary-adrenal (HPA) axis and increased sensitivity in the bilateral frontal lobes to perceived threats, leading to difficulties in emotional regulation, potential depression, and ultimately heightened suicidal ideation [80–82]. These findings underscore the urgent need for targeted interventions for bullying and emphasize the critical importance of promoting positive mental health development among adolescents.

### The moderating role of perceived family economic strain

The moderated mediation model employed in this study reveals that perceived family economic strain significantly moderates the indirect relationship between bullying victimization and depressive symptoms among both the general and minority adolescent populations. However, it fails to demonstrate a significant moderating influence of perceived family economic strain on either the direct path from bullying victimization to suicidal ideation or the indirect path from depressive symptoms to suicidal ideation. These findings suggest that the impact of bullying victimization on depressive symptoms may be mitigated by high levels of perceived family economic strain. Our findings contradict the prevalent notion that persistent financial stress within families exacerbates mental health problems in adolescents, as previously reported in research [83]. According to social comparison theory, an individual’s overall well-being is directly tied to their economic well-being, and financial stress can induce feelings of vulnerability and inadequacy [84, 85]. Similarly, Relative Deprivation Theory posits that individuals from less affluent backgrounds are more prone to feelings of deprivation, which may intensify psychological distress [86]. Interestingly, our results align with Jeong’s research, which proposes that adolescents from lower socioeconomic backgrounds may exhibit greater resilience to adverse events [87]. Several potential explanations can be offered for our findings. Firstly, China’s recent entry into a “well-off era” has significantly met the basic material needs of adolescents, prompting them to shift their focus from material goals to spiritual pursuits for greater psychological fulfillment. This paradigm shift may foster greater mental resilience, enabling them to cope with challenges such as bullying victimization. Furthermore, culturally-based factors significantly influence the moderating effect of perceived family economic strain on the relationship between bullying victimization and depressive symptoms among minority adolescents. Unique ethnic celebrations, such as the Fireworks Festival, Songkran Festival, and the Third Lunar Month Festival, reinforce cultural identity and community cohesion, which are linked to diminished stress levels and enhanced psychological resilience in youth [88]. Moreover, the abundance of fruits and vegetables in the Guangxi region, providing a vitamin-rich diet, may mitigate stress and promote mental and physical well-being [89]. The region’s natural environments, characterized by diverse green vegetation and picturesque landscapes, also contribute to mental wellness by alleviating stress [90].

When considered in the context of China’s economic and ethnic cultural background, the mental impact of perceived familial economic distress on adolescents may not be as severe as previously thought. It is crucial to adopt a holistic perspective, acknowledging the role of various systems in individual development. As general economic conditions improve, other psychological factors—such as parenting style, mental resilience, and academic pressure—may exert a more significant influence on mental health. Future research should delve deeper into the roles of these factors within moderated mediation models to better understand the intricate interplay between economic strain, cultural factors, and mental health outcomes in diverse populations.

### Strengths and limitations

A number of drawbacks are acknowledged in our work. First of all, self-reported statistics can be subjective. Personal prejudices may have affected the participants’ answers, leading them to respond according to how they understood the questions or what society expected of them. Future research should improve anonymity and combine self-reports with objective measurements like physiological evaluations or behavioral observations to improve data accuracy. Furthermore, our study’s cross-sectional design limits data collection to a single point in time, making it more difficult for us to prove causation. Our results should be viewed as showing connections rather than causation because we are unable to track the trajectory of changes in variables over a long period of time. Future longitudinal studies would be very beneficial since they would allow researchers to monitor the development of important variables over a number of time periods. This method would offer a more thorough and sophisticated comprehension of the ways in which suicidal ideation, depressive symptoms, and bullying victimization change and interact over time. Lastly, considering the regional character of our Guangxi sample, it is unclear whether our findings can be applied to other areas and nations. Guangxi’s distinct cultural and economic context may have an impact on these results. To gain a better understanding of teenage mental health difficulties in China, we recommend that future research employ a more representative sample from a wider geographic range.

Despite any limitations, the strength of this study lies in its large sample size of Chinese adolescents. Furthermore, an innovative aspect of this paper is the assessment of perceived family economic stress, rather than a sole reliance on objective measures of economic income. The broader implications of perceived family economic stress, including adolescents’ subjective perceptions, social comparisons, and life aspirations, allow us to gain a more comprehensive understanding of how family economic status affects individual mental health and social adjustment.Our results imply that depressive symptoms mediate the relationship between bullying victimization and suicidal ideation, while perceived family economic strain moderates the effect of bullying victimization on depressive symptoms. These revelations have important ramifications for public health initiatives. We support the introduction of thorough anti-bullying programs in schools, complete with educational curricula and social skills instruction meant to foster constructive peer relationships [91], as well as the development of strong monitoring and reporting mechanisms to assist bullied individuals. Additionally, we advocate for the provision of easily accessible mental health services for adolescent, with practitioners prepared to offer therapeutic methods like cognitive behavioral therapy to assist victims in managing the consequences of bullying. We urge parents to create an atmosphere at home that supports candid communication so that kids can freely talk about their school experiences and emotional health. Building their children’s resilience to overcome hardship is essential, and this includes giving them effective strategies to deal with and react to peer bullying, which is critical for adolescents’ mental health development.

## Conclusions

This study examined the role of depressive symptoms in mediating bullying victimization and suicidal ideation, as well as the moderating role of perceived family economic strain. Our findings confirm the hypotheses that bullying victimization may be positively associated with suicidal ideation. Furthermore, bullying victimization may indirectly influence suicidal ideation through the mediating role of depressive symptoms, and perceived family economic strain moderates the relationship between bullying victimization and depressive symptoms. These findings highlight the critical need to consider both depressive symptoms and broader socioeconomic contexts when addressing the impact of bullying on mental health.

## Electronic supplementary material

Below is the link to the electronic supplementary material.

Supplementary Material 1

Supplementary Material 2

Supplementary Material 3

Supplementary Material 4

## Data Availability

The datasets used and analysed during the study are available from the corresponding author on reasonable request.
