# Supplementary material for: Bullying victimization and suicidal ideation among Chinese adolescents: a moderated mediation model of depressive symptoms and perceived family economic strain
Source: BMC Public Health. 2025 Jan 30;25:393. doi: 10.1186/s12889-025-21579-w (PMC11783786; doi:10.1186/s12889-025-21579-w)
Supplement: Supplementary file 1 — Supplementary Material 1 [file 12889_2025_21579_MOESM1_ESM.pdf]

Personal Experiences Checklist – Short Form

Thinking about the last month or so at school, **how often** do the following things happen? Please **circle** the best response.

1.The other kids ignore me on purpose	Never	Rarely	Some-times	Most days or every day
2. Other kids make fun of my language	Never	Rarely	Some-times	Most days or every day
3. Other kids tease me about things that aren't true	Never	Rarely	Some-times	Most days or every day
4. Other kids threaten me over the phone	Never	Rarely	Some-times	Most days or every day
5. Other kids tell people not to hang around with me	Never	Rarely	Some-times	Most days or every day
6. Other kids won't talk to me because of where I'm from	Never	Rarely	Some-times	Most days or every day
7. Other kids say nasty things to me by texting	Never	Rarely	Some-times	Most days or every day
8. Other kids tell people to hit me	Never	Rarely	Some-times	Most days or every day
9. Other kids send me nasty emails	Never	Rarely	Some-times	Most days or every day
10. Other kids say mean things about me behind my back	Never	Rarely	Some-times	Most days or every day
11. Other kids shove me	Never	Rarely	Some-times	Most days or every day
12. Other kids say nasty things about me online	Never	Rarely	Some-times	Most days or every day
13. Other kids tell people to make fun of me	Never	Rarely	Some-times	Most days or every day
14. Other kids hit me	Never	Rarely	Some-times	Most days or every day
