# Supplementary material for: Bullying victimization and suicidal ideation among Chinese adolescents: a moderated mediation model of depressive symptoms and perceived family economic strain
Source: BMC Public Health. 2025 Jan 30;25:393. doi: 10.1186/s12889-025-21579-w (PMC11783786; doi:10.1186/s12889-025-21579-w)
Supplement: Supplementary file 2 — Supplementary Material 2 [file 12889_2025_21579_MOESM2_ESM.pdf]

条目	从不	有时	经常	几乎总是
V1. 故意无视我的存在	1	2	3	4
V2. 用不真实的事情取笑我	1	2	3	4
V3. 告诉其他同学不要和我一起玩	1	2	3	4
V4. 在背后说我坏话	1	2	3	4
V5. 让其他同学联合起来取笑我	1	2	3	4
V6. 因口音或语言（如夹壮、说壮话）取笑我	1	2	3	4
V7. 因为我来自哪里（如农村）而排挤我	1	2	3	4
V8. 在电话/微信/QQ 里威胁我	1	2	3	4
V9. 通过短信/微信/QQ 对我说一些肮脏的话	1	2	3	4
V10. 给我发了一些令人讨厌的话或短视频	1	2	3	4
V11. 在朋友圈/说说/抖音/快手散布关于我的坏话	1	2	3	4
V12. 告诉其他同学，让他/她们一起打我	1	2	3	4
V13. 猛推/乱撞我	1	2	3	4
V14. 打我的身子，如头部	1	2	3	4

Items	Never	Sometimes	Often	Almost always
V1. Deliberately ignoring my existence	1	2	3	4
V2. Making fun of me with things that aren’t true	1	2	3	4
V3. Tell other students not to play with me	1	2	3	4
V4. Talking behind my back	1	2	3	4
V5. Let the other students gang up and make fun of me	1	2	3	4
V6. Making fun of me because of my accent or language (e.g., talking strong)	1	2	3	4
V7. Exclusion of me because of where I am from (e.g. rural)	1	2	3	4
V8. Threatening me on the phone/wechat /QQ	1	2	3	4
V9. Say dirty things to me via messages /wechat /QQ	1	2	3	4
V10. Sent me some annoying words or short videos	1	2	3	4
V11. Spreading bad things about me in moments/Talk/Tik Tok/Quick hand	1	2	3	4
V12. Tell the other students to let him/them hit me	1	2	3	4
V13. Shove/bump me	1	2	3	4
V14. Hit me on the body, like the head	1	2	3	4
