# Supplementary material for: Bullying victimization and suicidal ideation among Chinese adolescents: a moderated mediation model of depressive symptoms and perceived family economic strain
Source: BMC Public Health. 2025 Jan 30;25:393. doi: 10.1186/s12889-025-21579-w (PMC11783786; doi:10.1186/s12889-025-21579-w)
Supplement: Supplementary file 3 — Supplementary Material 3 [file 12889_2025_21579_MOESM3_ESM.pdf]

Check for updates

Original Research

Journal of Interpersonal Violence
1–21
© The Author(s) 2019
Article reuse guidelines:
sagepub.com/journals-permissions
DOI: 10.1177/0886260519875559
journals.sagepub.com/home/jiv
SAGE

Construction and Psychometric Properties of a Short Form of the Personal Experiences Checklist

Karoline Prinz,[1] Daniel Costa,[1] Elizabeth Chervonsky,[1] and Caroline Hunt[1]

Abstract

Peer victimization is a significant problem concerning young people. However, there are few brief measures that cover the key domains of victimization, including cyber victimization. Therefore, the objective of the present study was to construct and validate a short form version of the Personal Experiences Checklist (PECK) to be able to measure victimization efficiently and effectively. The PECK was originally developed to measure a young person's experience of victimization and provides scores for four subscales (relational-verbal, cyber, physical, and victimization in relation to culture) as well as a total score. A sample of N = 1,040 school students from Grades 4 to 9 (mean age = 12.2 years) derived from two studies was used to construct and cross-validate the structure of a Personal Experiences Checklist Short Form (PECK-SF) using confirmatory factor analysis and item response theory analysis. Based on discriminative ability and consideration of item content, 14 items were selected to form the PECK-SF, which demonstrated good internal consistency (Cronbach's α = .83). Given the small number of items, the use of subscales is not advised. However the PECK-SF total scale represents a potential alternative to the long form of the PECK and provides a brief and general index of victimization.

[1]The University of Sydney, Camperdown, New South Wales, Australia

Corresponding Author:
Caroline Hunt, School of Psychology, Brain and Mind Centre, The University of Sydney, 94 Mallett Street, Camperdown, New South Wales 2056, Australia.
Email: caroline.hunt@sydney.edu.au

Keywords
bullying, Internet and abuse, mental health and violence

Bullying and victimization are long recognized problems that affect youth. Children and adolescents report a range of comorbid problems associated with bullying, including anxiety and depression (Cook, Williams, Guerra, Kim, & Sadek, 2010; Reijntjes, Kamphuis, Prinzie, & Telch, 2010), low self-esteem and self-compassion (Cook et al., 2010), psychosomatic complaints (Fekkes, Pijpers, Fredriks, Vogels, & Verloove-Vanhorick, 2006), impact on academic performance (Vaillancourt, Brittain, McDougall, & Duku, 2013), and suicide ideation (Kowalski, Giumetti, Schroeder, & Lattanner, 2014). Longitudinal studies have shown that experiencing victimization during school years predicts mood disorders and suicide into adulthood (Klomek, Marrocco, Kleinman, Schonfeld, & Gould, 2007). Furthermore, early involvement in bullying tends to manifest in continued experiences of victimization across time (Barker et al., 2008).

Despite advances in our understanding of bullying and the development of interventions, bullying remains common in schools. For example, the World Health Organization (2002) have released statistics on the high proportion of 13-year-old children in many countries who experience bullying behavior in school in a typical week (e.g., 7.6% USA, 7.3% Canada, 9.6% Denmark). Furthermore, a prevalence study conducted by the Child Health Promotion Research Center (Cross et al., 2009) has shown that approximately one in four (27%) Australian students (in Grades 4 to 9) experience victimization every few weeks.

Given the significance of the problem, it is critical to be able to measure victimization and bullying accurately and efficiently. Accurate measurement of bullying requires assessment of its specific characteristics of intent, repetition, and the context of a power imbalance (Jia & Mikami, 2018). Peer victimization, on the contrary, which is the focus of this article, is a broader construct and defined as the experience of being a target of the aggressive behavior of other children (e.g., Hawker & Boulton, 2000). Victimization can take many forms, and a comprehensive measure will need to cover these key forms. Recognized forms include direct verbal and physical forms of victimization where the aggressor is known (e.g., name calling, hitting or kicking), indirect or relational victimization where the aggressor may not be known (e.g., social exclusion, spreading rumors), and victimization using new technology (cybervictimization) (Menesini & Salmivalli, 2017). It has been suggested that the ideal assessment of victimization uses a multi-informant approach by taking into account the observations of peers, teachers, and

parents, as well as self-report (Demaray, Malecki, Secord, & Lyell, 2013; Rupp, Elliott, & Gresham, 2018).

Although a multi-informant approach has advantages, self-reports access unique aspects of the personal experience of victimization. Empirical studies on the concordance between self- and peer reports have concluded that the measures tap into different aspects of victimization. For example, Pouwels, Lansu, and Cillessen (2016) found a small but significant correlation of $r = .23$ ($p < .01$) between self- and peer reports in an adolescent sample. Schäfer, Werner, and Crick (2002) concluded that the difference between self- and peer reports of victimization was that self-report reflects a subjective experience of victimization, while peer nominations rated experience relative to peers.

Each assessment approach has both advantages and disadvantages. Although peer nominations reduce the influence of individual bias and increase reliability (Marks, Babcock, Cillessen, & Crick, 2013), some children may be nominated not because of observed behaviors but instead because of their classroom reputation (e.g., Scholte, Burk, & Overbeck, 2013). Furthermore, the use of peer, parent, and teacher nominations include the significant resource demands and findings that bullying is often unreported to teachers (e.g., Hunt, 2007) or is often unobserved by teachers or peers (e.g., Schäfer et al., 2002). Rupp et al. (2018) assessed cross-informant consistency for student, parent, and teacher reports of bullying behavior in a sample of elementary school students. Although the informants were significantly consistent in their reports, with correlations ranging from $r = .43$ to .55, students reported higher scores than parents and teachers, suggesting that adult informants may underestimate bullying behavior.

In sum, the self-report of victimization experiences provides a crucial view of the extent and impact of victimization on its targets, particularly those forms that may not be apparent to teachers, parents, or peers. Self-report further provides a unique and essential perspective of a young person's own experience of victimization (Hunt, Peters, & Rapee, 2012). Indeed, self-reported victimization has been found to be associated with self-reported peer-related problems (social anxiety, loneliness) and emotional problems (self-esteem, depressive symptoms) while controlling for peer-reported victimization (Pouwels et al., 2016). A further advantage of self-report, particularly the use of shortened measures, is its use as an efficient and effective screen to identify youth for a more resource intensive multi-informant assessment.

Despite the growing number of self-reported victimization measures published, there are few that meet all research requirements, and few that contain items across its different forms. For example, a recent systematic review of bullying assessments published prior to 2010 concluded that only half the assessments featured cyber bullying and of those, there was limited evidence

of validity and reliability (Berne et al., 2013). Furthermore, brief instruments that contain items targeting multiple forms of victimization are lacking. For example, current measures that have between six and 14 items (e.g., the Peer Victimization Scale, Austin & Joseph, 1996; the California Bullying Victimization Scale, Felix, Sharkey, Green, Furlong, & Tanigwa, 2011; the Gatehouse Bullying Scale, Bond, Wolfe, Tollit, Butler, & Patton, 2007; the Peer Victimization Scale, Callaghan & Joseph, 1995; the Perception of Teasing Scale, Jensen & Steele, 2010; and the Physical Appearance Related Teasing Scale, Vessey, Duffy, O'Sullivan, & Swanson, 2003) do not assess cyber victimization. Therefore, there is a need for the development of an economical and time efficient assessment that provides a broad-based general index of victimization. This is particularly relevant to the selection of measures of victimization in educational and clinical settings, where ease and length of administration may be a critical factor. Short forms of assessment can also provide a screen to identify children who are likely to have been bullied, and who can then be selected for an assessment that is more comprehensive.

The Personal Experiences Checklist

The original version of the Personal Experiences Checklist (PECK) was developed to provide a dimensional assessment of a young person's experience (aged 9-16 years and above) of direct or indirect victimization (Hunt et al., 2012). Hunt and colleagues' (2012) aim was to provide a comprehensive self-report measure assessing specific victimization behaviors. Thus the measure was behaviorally operationalized to reduce interpretation bias, given evidence that young people of different ages and from different cultures can vary in their understanding of the construct of bullying (e.g., Boulton, Bucci, & Hawker, 1999; Smith, Cowie, Olafsson, & Liefooghe, 2002). Initial items were developed based on a search of the literature and existing measures, feedback from experts in the field of bullying, and subjected to item and factor structure analyses. Four factors that assessed relational-verbal victimization, physical victimization, cyber victimization, as well as items that represented victimization related to culture were established to form the 32 items scale (shown in Table 1), and were supported in a second sample using confirmatory factor analysis (CFA). CFA also supported a higher order victimization factor with direct effects on these four factors. The PECK demonstrated good internal consistency (Cronbach's α range = .78-.91) and adequate test–retest reliability (range r = .61-.86). Evidence for validity of the PECK included associations with peer nominated assessments of victimization, existing self-report assessment of victimization, and self-report anxiety, depression, and self-esteem.

Table 1. Items of the Personal Experiences Checklist (PECK) and the PECK Short Form (PECK-SF).

PECK	PECK-SF	Item Wording	Subscale
1		Other kids play nasty practical jokes on me	Physical
2	1	The other kids ignore me on purpose	Relational verbal
3		Other kids try to turn my friends against me	Relational verbal
4		Other kids say nasty things to me on an instant messenger, chat room, or bulletin board	Cyber
5	2	Other kids make fun of my language	Culture
6	3	Other kids tease me about things that aren't true	Relational verbal
7		Other kids punch me	Physical
8		Other kids make fun of my culture	Culture
9		Other kids make prank calls to me	Cyber
10	4	Other kids threaten me over the phone	Cyber
11	5	Other kids tell people not to hang around with me	Relational verbal
12	6	Other kids won't talk to me because of where I'm from	Culture
13		Other kids make death stares at me	Relational verbal
14	7	Other kids say nasty things to me by text messages	Cyber
15	8	Other kids tell people to hit me	Physical
16	9	Other kids send me nasty emails	Cyber
17		Other kids kick me	Physical
18	10	Other kids say mean things about me behind my back	Relational verbal
19		Other kids make rude gestures at me	Relational verbal
20		Other kids say they'll hurt me if I don't do things for them	Physical
21	11	Other kids shove me	Physical
22	12	Other kids say nasty things about me online	Cyber
23		Other kids wreck my things	Physical
24		Other kids send me computer viruses on purpose	Cyber
25		Other kids tease me about my voice	Culture
26		Other kids trip me over	Physical

(continued)

Table 1. (continued)

PECK	PECK-SF	Item Wording	Subscale
27	13	Other kids tell people to make fun of me	Relational verbal
28		Other kids call me names because I'm a bit different	Relational verbal
29	14	Other kids hit me	Physical
30		Other kids harass me over the phone	Cyber
31		Other kids make fun of my friends	Relational verbal
32		Other kids call me names because I can't do something	Relational verbal

Note. PECK = Personal Experiences Checklist; PECK-SF = Personal Experiences Checklist-Short Form.

The aim of the current article was to use a community sample to develop a PECK Short Form (PECK-SF) with strong psychometric properties across the same key forms of victimization as the original instrument, for use in settings where the need for a quick assessment or screening measure is critical. As with the original PECK, the items operationalize typical bullying behaviors, yet the measure does not assess specific bullying characteristic of intent, repetition, or the context of a power imbalance. As the PECK-SF does not capture the defining aspects of bullying, it is a measure of peer victimization. The aim of the current article is to describe the development of a short form of the PECK, using CFA and item response theory (IRT). We intended that the PECK-SF would discriminate between groups of students who had identified themselves as having either experienced significant victimization or not on an established self-report measure, as well as between groups of students who had been identified by their peers as experiencing significant victimization or not. A further aim was to examine the short form questionnaire's functioning concerning diversity, specifically gender and the self-reported ethnic identification of students.

Method

Participants

All participants from two existing data sets were pooled into a combined sample ($N = 1{,}040$). The first group of participants ($n = 192$) completed the PECK as part of a larger study (Chervonsky & Hunt, 2019) which assessed the role of emotional regulation strategies in social relationships. Seventy-three percent ($n = 140$) of this sample had peer nomination data. The second

participant group (n = 848) was a subset of the data from 14 schools used in the initial validation study of the original PECK (Hunt et al., 2012). Thirty percent (n = 218) of this sample had peer nomination data. The University of Sydney Human Research Ethics Committee approved both studies. Informed consent was obtained from all participating students included in the study and one of their parents. Participants with peer nomination data were selected from the combined sample and set aside to perform the validation analyses (N = 358, validation sample). Remaining data from the combined sample, that is, participants who did not complete a peer nomination process because their school did not agree to this component of the assessment, were analyzed to derive the short scale items (N = 682, scale development sample).

Across the combined sample (N = 1,040), 53.3% were female and the mean age was 12.2 years (SD = 1.5). The student self-identified their ethnic background, with the most common groups being Caucasian (61.5%), East Asian (13.7%), Mediterranean (4.7%), and Middle Eastern (4.4%). They were recruited from Grades 4 to 9 across 19 schools. In the scale development sample (n = 682), there were 55.3% female students and the mean age was 12.4 years (SD 1.6). The most common self-identified ethnicity groups were Caucasian (54.9%), East Asian (15.7%), Mediterranean (7.8%), and Middle Eastern (7.8%). In the validation sample (n = 358), 49.4% were female and the mean age was 11.9 years (SD = 1.3). The most common self-identified ethnicity groups were Caucasian (62.5%), East Asian (13.4%), West Asian (4.8%), and Mediterranean (4.2%).

Test–retest analyses were conducted using the subsample of participants reported in the original PECK development article, with a retest interval of 2 weeks (Hunt et al., 2012) (n = 78; 46.2% female; mean age 12.0 years, SD = 0.9). This sample of children mostly self-identified as Caucasian (57.1%), Mediterranean (14.3%), and East Asian (12.5%).

Measures

Personal Experiences Checklist (PECK). The PECK (Hunt et al., 2012) is a self-report assessment of the experience of victimization that provides a score on four subscales (verbal-relational, physical, cyber, and victimization related to culture) as well as a total scale score. Respondents assess each behavior on a 5-point scale (*never, rarely, sometimes, most days, every day*), with higher scores representing higher levels of victimization.

Peer nomination. Peer nominations were collected using standard procedures (Coie, Dodge, & Coppotelli, 1982). Participants were provided with a list of those students in their grade with consent to participate in the research, and asked to nominate up to three “people in your grade who best match the

descriptions" for three victimization items that were developed by the authors ("kids who get hit, kicked or pushed around a lot"; "kids who get called names, laughed at, or made fun of a lot"; "kids who get talked about behind their backs, are ignored, or left out of things a lot"). The items were embedded among a number of nonaggressive behaviors (e.g., "kids who are good at sport"; "kids who will help you if you are in trouble"; "kids you do not like a lot"). Scores were converted into z scores within each grade to account for different grade sizes.

Olweus Bully/Victim Questionnaire (OBVQ). The OBVQ (Olweus, 1996) is an established self-report bullying measure. The items used in the current study were global (how often have you been bullied?), verbal (called mean names, made fun of, teased in hurtful way), being excluded (left out on purpose, excluded, ignored), physical (hit, kicked, pushed shoved around, locked indoors), and having rumors spread (lies or false rumors spread about me made others dislike me). Participants are first given a definition of bullying before rating the frequency of victimization. The current study used the recommended response cut-point of "2 or 3 times a month" to indicate significant experiences of victimization (Solberg & Olweus, 2003). In the current study, the items were not aggregated but treated as individual one-item scales. There is evidence of modest support for concurrent validity of the global victimization item (Lee & Cornell, 2009). Using the Solberg and Olweus standard cut-point, middle school students identified as victims had significantly more peer nominations (Cohen's $d = .76$, $p < .01$) than nonvictims, providing support of concurrent validity. In terms of convergent validity, students identified as victims had lower academic grades (Cohen's $d = -.50$, $p < .05$) than nonvictims (Lee & Cornell, 2009).

Analyses

CFA is a correlation-based method of empirically testing a hypothesized factor structure, in which observed variables are used to represent unobserved (latent) variables. The established factor structure of the PECK was tested for the current sample using CFA in MPlus Version 6 (Muthén & Muthén, 2011); namely, the four subscales (relational-verbal victimization, physical victimization, cyber victimization, and victimization related to culture) were modeled using their respective items and were posited to load on a higher order victimization factor. The mean- and variance-adjusted weighted least squares estimation procedure was employed because the item responses of the PECK are ordinal. The following model fit indices (and commonly accepted criteria for indicating

good model fit; Hu & Bentler, 1999) were examined χ^2 test (nonsignificant), the root mean square error of approximation (RMSEA, < 0.06, with a 90% confidence interval [CI] including 0.05), comparative fit index (CFI, > 0.95), and Tucker–Lewis index (TLI, > 0.95).

IRT posits that the probability of an individual's response on an item is determined by their value on the latent variable and properties of the item. Samejima's (1969) two-parameter graded response model was employed, in which the observed responses to polytomous items (i.e., items with more than two response options) are assumed to reflect an underlying latent variable, such that the probability of responding with a higher response option increases as the level of the latent variable increases. The two types of item parameters estimated for each item were (a) difficulty, or location along the continuum of values of the latent trait; and (b) discrimination, or ability to differentiate between those scoring high and low on each victimization domain represented in the PECK. This was done to ensure that the short form retained coverage of the broad range of bullying behaviors identified in the development of the PECK. The analysis was conducted using the grm function of the ltm package (Rizopoulos, 2006) in R and was performed separately for the factors identified using CFA (i.e., unidimensional models). The aim of this analysis was to examine (a) the item characteristic curve for each item, focusing on problematic item response category thresholds (i.e., the value of the latent variable for which adjacent response categories are equally likely); and (b) the item discrimination parameters, which describe the ability of each item to discriminate between individuals scoring low and high on the latent variable. Problems with threshold ordering suggest that respondents are not using the response scale in the manner expected.

Selection of short form items. Items were selected for the short form primarily based on the magnitude of their discrimination parameters, but also based on consideration of their content, ensuring that the four forms of victimization were covered. Furthermore, if a pair of items exhibited a large residual correlation, only one of the items—that with the largest discrimination parameter—was included.

Test–retest reliability and validation analyses. An intraclass correlation was used to calculate test–retest reliability, using a 2-way mixed effects model with absolute agreement. For the analysis of the validation data set ($N = 358$), only those items provisionally selected to constitute the short form were included, and the validation analysis included any modifications suggested by the initial CFA and IRT.

Results

Preliminary analyses indicated that there were no significant differences between the sample with peer nomination data and those without in terms of reported victimization experience on the Personal Experiences Checklist total scale score (t = 0.61, df = 1038, p = .54) or gender (χ^2 = 3.21, p = .7). However, those students with peer nomination data (mean age = 11.9 years, SD = 1.3) were younger than those students with no peer nomination data (mean age = 12.4 years, SD = 1.6) (t = 4.85, df = 1038, p < .001).

The item response frequencies for the initial data set (N = 682) are shown in Table 2. The standardized factor loadings from the CFA are also reported in Table 2. The fit indices for this model were χ^2 = 1,128.26 (p < .001), RMSEA = 0.046 (90% CI [0.043, 0.050]), CFI = 0.94, and TLI = 0.94. Large residual correlations were observed between three pairs of items: 7 with 29, 3 with 11, and 5 with 8. The modeling of these covariances was considered theoretically defensible as the items within each pair overlapped in content (e.g., Item 7: "other kids punch me" and Item 29: "other kids hit me"), and the model fit improved to χ^2 = 949.11 (p < .001), RMSEA = 0.040 (90% CI [0.036, 0.043]), CFI = 0.96 and TLI = 0.96. In light of known sensitivity of the χ^2 statistic to sample size and the magnitude of the other fit indices observed, we considered the model to fit the data well.

Results of the IRT analysis are shown in Table 2. Note that Items 5 and 15 could not be analyzed because one of the middle categories (in both cases "Most days") had zero frequency. All items exhibited the same problematic item response thresholds, namely that the fourth category ("Most days") did not have the highest probability for any value of the latent variable; see Figure 1 for an example item characteristic curve. This result aligned with two characteristics of the item response frequencies (see Table 2): (a) the fourth ("Most days") and fifth ("Every day") response categories tended to have very low frequencies, particularly for the cyber victimization domain; and (b) for 20 of the 32 items, the fourth category had a lower frequency than the two adjacent categories. Three items (4, 8, and 28) also exhibited a problem with the second category ("Rarely"; see Figure 2). These problematic item response thresholds and low frequencies suggest that "Most days" and "Every day" were not both necessary, and would benefit from being combined into a single category. This was done by recoding the fourth and fifth response categories as a single category, and relabeling this category "most days or every day." This rescoring process was carried out for all 32 items and the IRT analysis was rerun. Results demonstrated that the location and discrimination parameters were very similar, with the exception that the previously excluded Items 5 and 15 could be included. The rescored Item 5 had the highest discrimination parameter in the culture domain,

Table 2. Item Response Frequencies and Results of the CFA and IRT Analysis.

Item	Frequency					CFA	IRT				
	Never	Rarely	Sometimes	Most Days	Every Day	Loadings[a]	Thresholds	Discrimination			
Relational verbal											
2	295	235	120	17	15	0.65	−0.28	1.07	2.48	3.01	1.61
3	370	169	110	12	21	0.71	0.12	1.05	2.35	2.67	1.79
6	288	212	125	29	28	0.74	−0.29	0.76	1.81	2.32	2.08
11	476	122	56	14	14	0.79	0.61	1.39	2.20	2.62	2.33
13	492	121	40	14	15	0.65	0.84	1.89	2.67	3.21	1.50
18	211	243	166	29	33	0.79	−0.62	0.47	1.56	2.00	2.64
19	418	151	74	22	17	0.77	0.37	1.31	2.24	2.88	1.76
27	556	76	36	8	6	0.87	1.00	1.68	2.50	2.97	2.66
28	455	98	75	16	38	0.64	0.67	1.39	2.31	2.65	1.34
31	282	198	158	16	28	0.55	−0.44	0.97	2.92	3.43	1.07
32	447	131	75	12	17	0.75	0.51	1.36	2.44	2.84	1.75
Cyber											
4	521	82	66	8	5	0.67	1.03	1.72	3.10	3.71	1.67
9	473	128	61	6	14	0.63	0.90	2.07	3.43	3.75	1.22
10	649	19	11	2	1	0.93	1.90	2.31	2.91	3.17	3.32
14	613	49	18	1	1	0.57	1.77	2.68	4.02	4.41	1.83
16	585	71	23	2	1	0.68	1.37	2.25	3.38	3.89	2.32
22	641	33	7	1	0	0.77	2.00	2.91	3.88	-	2.31
24	636	31	10	3	2	0.71	2.15	2.93	3.61	4.16	1.77
30	644	27	9	1	1	0.87	1.93	2.59	3.32	3.60	2.70
Physical											
1	387	215	62	7	11	0.74	0.26	1.92	3.25	3.72	1.33
7	403	161	84	15	19	0.72	0.31	1.21	2.16	2.53	2.16
15	586	58	32	0	6	0.82	-	-	-	-	-
17	518	110	40	6	8	0.83	0.79	1.53	2.34	2.69	3.45
20	556	74	36	4	12	0.80	1.22	1.97	2.86	3.05	1.79
21	436	139	72	15	20	0.74	0.46	1.28	2.14	2.50	2.16
23	444	154	70	10	4	0.74	0.56	1.80	3.33	4.17	1.43
26	489	134	46	4	9	0.77	0.71	1.64	2.61	2.84	2.46
29	487	111	55	13	16	0.79	0.66	1.34	1.96	2.26	3.00
Culture											
5	559	68	45	0	10	0.62	—	—	—	—	—
8	531	75	62	5	9	0.66	1.09	1.73	2.93	3.21	1.76
12	644	20	14	1	3	0.95	1.80	2.23	2.89	3.01	3.33
25	542	83	35	10	12	0.77	1.65	2.83	3.90	4.58	0.96
PECK total											
Relational verbal						1.00					
Cyber						0.64					
Physical						0.67					
Culture						0.65					

Note. CFA = confirmatory factor analysis; IRT = item response theory; PECK = Personal Experiences Checklist.
[a]Standardized factor loadings.

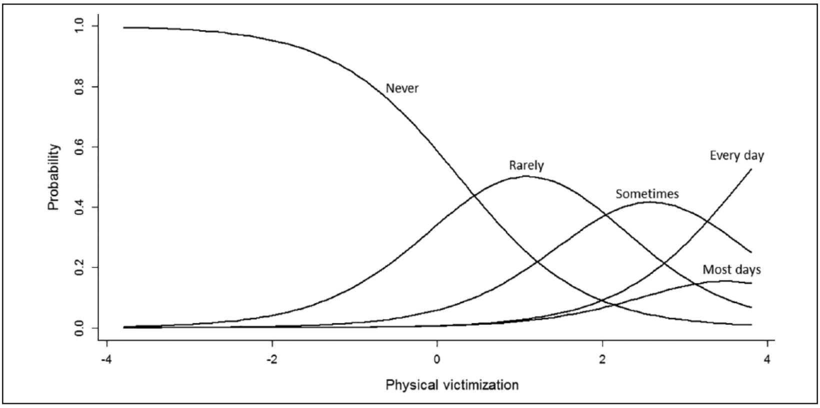

Figure 1. Item characteristic curve for Item 1.
Note. The probability of each response category is plotted against the level of the latent variable (in this case, physical victimization). Note that the fourth response category ("Most days") does not have the highest probability for any value of the latent trait.

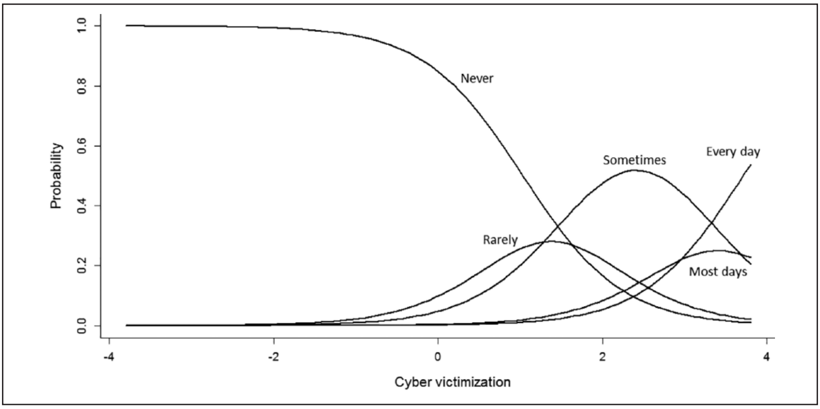

Figure 2. Item characteristic curve for Item 4.
Note. The second ("Rarely") and fourth ("Most days") response categories do not have the highest probability for any value of the latent variable (in this case, cyber victimization).

and Item 15 had the fourth highest discrimination parameter in the physical victimization domain.

Based on these results, a preliminary short form version of the instrument was developed (see items in Table 1). Items were chosen primarily because of their discrimination, but content was also taken into account. For example,

Table 3. Item Response Frequencies and Results of the CFA and IRT Analysis on the Short Form Instrument.

	Frequency				CFA	IRT			
Item	Never	Rarely	Sometimes	Most Days/ Every Day	Loadings[a]	Thresholds	Discrimination		
Relational verbal									
2	154	133	55	16	0.73	−0.23	1.12	2.26	2.04
6	142	118	73	25	0.82	−0.31	0.72	1.80	2.58
11	219	89	37	13	0.85	0.33	1.22	2.06	3.33
18	113	114	95	36	0.77	−0.62	0.44	1.66	2.13
27	272	64	20	2	0.88	0.85	1.85	3.14	2.53
Cyber									
10	344	12	2	0	0.53	2.875	4.37	—	1.39
14	319	28	11	0	0.60	1.614	2.50	—	2.04
16	312	36	9	1	0.84	1.566	2.72	4.15	1.82
22	328	22	8	0	0.76	1.812	2.69	—	2.08
Physical									
15	286	55	10	7	0.89	0.97	1.90	2.39	2.90
21	198	106	39	15	0.79	0.19	1.32	2.17	2.25
29	251	67	31	9	0.78	0.61	1.37	2.19	3.23
Culture[b]									
5	285	40	19	14	0.66	1.031	1.66	2.22	2.22
12	324	28	4	2	0.93	1.646	2.70	3.26	3.26
PECK total									
Relational verbal					0.96				
Cyber					0.58				
Physical					0.77				
Culture					0.72				

Note. CFA = confirmatory factor analysis; IRT = item response theory; PECK = Personal Experiences Checklist.
[a]Standardized factor loadings.
[b]A two-parameter IRT model could not be conducted on this two-item domain, so the IRT parameters reported are for a one-parameter model (i.e., with discrimination held constant across the items).

five items with the highest discrimination were selected from the relational victimization domain, with the exception of "other kids try to turn my friends against me," due to its similar content with the higher discriminating item "other kids tell people not to hang around with me." Similarly, "other kids harass me over the phone" was not selected as it was less discriminating than an item with similar content "other kids threaten me over the phone." With the last two response options combined, the CFA and IRT analysis were rerun on the 14 short form items in the validation data set (Table 3).

The standardized factor loadings from the CFA conducted on the short form are reported in Table 3. The fit of this model was good: $\chi^2 = 104.12$, $p = .006$; RMSEA = 0.036, 90% CI [0.020, 0.050]; CFI = 0.99 and TLI = 0.99. Results of the IRT are also shown in Table 3. In spite of the rescoring, three of the four cyber victimization items still had zero frequency for the last ("Most days/Every day") category, and so only two threshold parameters could be estimated for these items. Also note that a two-parameter IRT model could not be conducted on the two-item *culture* domain, so a one-parameter model was used instead (i.e., with discrimination held constant across the items). None of the item characteristic curves for the 14 items exhibited threshold problems, and the discrimination parameters did not differ greatly between items within a domain.

Item Scale Characteristics

The internal consistency of the PECK-SF was assessed for both samples. In the initial data set ($N = 682$), a Cronbach's α of .82 was found for the full PECK-SF scale, $\alpha = .82$ for the relational-verbal victimization items, $\alpha = .63$ for the cyber victimization items, $\alpha = .68$ for the physical victimization items, and $\alpha = .48$ for the victimization based on culture items. The average inter-item correlation for each scale was $r = .24$ for the full PECK-SF, $r = .48$ for relational-verbal victimization, $r = .31$ for cyber victimization, $r = .43$ for physical victimization, and $r = .36$ for victimization based on culture.

In the validation data set ($N = 358$), a Cronbach's α of .84 was found for the full PECK-SF scale, $\alpha = .84$ for the relational-verbal victimization items, $\alpha = .57$ for the cyber victimization items, $\alpha = .75$ for the physical victimization items, and $\alpha = .49$ for the victimization based on culture items. The average interitem correlation for each scale was $r = .25$ for the full PECK-SF, $r = .53$ for relational-verbal victimization, $r = .25$ for cyber victimization, $r = .51$ for physical victimization, and $r = .39$ for victimization based on culture. Given the item scale characteristics did not support the use of subscales, validity evidence and test–retest reliability is provided for the total scale score only.

Concerning test–retest reliability, the intraclass coefficient for the PECK-SF was .79 (95% CI [.70, .86]) ($p < .001$).

Validity Evidence Based on the Relation of the PECK-SF to Other Variables

Validity evidence, reported in Table 4, was based on the $N = 358$ sample where peer nomination and additional self-report data were available. The PECK-SF total score was found to significantly differentiate those students

Table 4. Mean (and Standard Deviation) Scores on PECK-SF Total Score for Young People With Significant Reports of Being Victimized Based on OBVQ or By Peer Nomination.

OBVQ Cut-Points	Significant Victimization	No Significant Victimization	*t*	*p*
Global	12.47 (6.02)	4.95 (4.70)	−10.42	< .001
Verbal	12.98 (6.09)	5.15 (4.83)	−9.74	< .001
Excluded	13.18 (5.77)	5.22 (4.93)	−9.43	< .001
Physical	13.15 (7.22)	5.56 (5.09)	−7.09	< .001
Rumors	14.38 (6.01)	5.38 (4.97)	−9.19	< .001
Peer nominations: hit, kicked, or punched	8.88 (7.29)	5.73 (5.24)	−3.51	.001
Peer nominations: called names or laughed at	9.93 (7.57)	5.76 (5.28)	−3.98	<.001
Peer nominations: talked about, ignored, or left out	9.85 (7.44)	5.65 (5.18)	−4.52	< .001

Note. PECK-SF = Personal Experiences Checklist Short Form; OBVQ = Olweus Bully/Victim Questionnaire.

who were identified as having experienced significant victimization, both on self-report using the OBVQ (global, verbal, excluded, physical, and rumors items) and through peer nomination (physical, verbal, and relational victimization), from those students identified as not experiencing significant victimization. The PECK-SF was compared with the PECK long form, resulting in a Pearson correlation of $r = 0.953$ ($p < .001$).

Differences in PECK-SF Scores Related to Gender and Self-Identified Ethnicity

Group comparisons using PECK-SF total scores were conducted for female versus male students, and for those students who self-identified their ethnic identity as Anglo-Australian (the majority culture in participating schools) versus those who self-identified with other minority cultural groups. Independent *t* tests indicated no mean score differences in terms of gender ($t = 1.69$, $df = 1038$, $p = .090$) or ethnic majority/minority status ($t = -1.07$, $df = 1038$, $p = .285$).

Discussion

Using IRT, 14 items were selected that best discriminated between individuals' scores within each of the four factors identified in the original 32-item

PECK, as well as providing a broad coverage as possible across content areas of the four forms of victimization: relational-verbal, physical, and cyber victimization, and victimization based on culture. Confirmatory factor analyses indicated that the four-factor structure for both the original and the short form of the PECK was a good fit of the data. However, despite the four-factor structure being supported, the cyber victimization subscale had questionable internal consistency and the victimization based on culture subscale had unacceptable internal consistency, likely due to the small number of items within these scales. Only the use of the full scale as a general index of victimization is therefore recommended.

A further modification to the PECK-SF was the reduction of the number of response options. The item response frequencies for the last two categories ("Every day" and "Most days") were very low for most of the items and in some cases were zero, illustrating that in this sample certain types of victimization occur with relatively low frequency. The IRT analysis supported the collapsing of these two categories, and further confirmation of these low frequencies supports combining these two categories into a single category ("Most days or every day").

In terms of validity evidence, the 14-item PECK-SF successfully discriminated between young people who had self-identified as experiencing significant victimization using the OBVQ, a measure that takes a different approach to assessment by first providing a definition of bullying, relative to those young people who did not report significant victimization. Not imposing a specific definition of bullying can be viewed as a strength of the PECK-SF, as bullying can mean different things to different people. However, for this reason, the scale is best considered as a measure of victimization as it does not assess specific bullying characteristic of intent, repetition, or the context of a power imbalance (Jia & Mikami, 2018). The PECK-SF also discriminated between those who were identified by their peers as being subjected to physical, verbal, or relational victimization, and those who were not. A significant correspondence between the long and short forms of the PECK provides further validity evidence.

These findings provide preliminary support for the structure of the PECK-SF; however, there is a need to replicate further the findings in a new sample to address the limitations of the current study, including the fact that a majority of the data were used for validity evidence for the original long form version of the PECK. Furthermore, using data from two previous samples, collected several years apart, adds to the need to replicate the results. The sample was split according to the existence of peer nomination data due to a number of schools declining to have peer nomination in the assessment protocol. While the two subsamples were equivalent in terms of gender and

level of self-reported victimization, those students without peer nomination data were older, consistent with our experience that sensitivities to the use of peer nomination do appear to rise with the age of students. Unfortunately, the coding of the peer nomination data did not allow the internal reliability for this assessment to be calculated. This lack of psychometric support for the peer nomination items is a limitation of the validity evidence.

There are a number of additional limitations for consideration. Our analyses assumed homogeneity in measurement properties across schools and did not account for potential clustering. Given potential differences across schools in levels of victimization, multilevel analyses would have been desirable. Regrettably, this was not possible with the current data due to failed model convergence, likely due to the combination of the large number of items, the modeling of ordinal data, and the relatively small number of clusters. The current lack of evidence to support the convergent and discriminant validity of the measure prevents any firm conclusion to be reached about the utility of the PECK-SF beyond existing scales until this requirement is addressed in future research, although the present results provide clear evidence of its internal structure. Furthermore, given the problematic item characteristics of the subscales, and the likely loss of precision in using scales with a very small number of items, it is recommended that researchers use only the full scale of the PECK-SF as a general continuous index of the main forms of victimization.

Consistent with other published Australian data (Nguy & Hunt, 2004), the PECK-SF, as a broadly focused assessment of victimization, did not reflect any cultural differences in terms of ethnic majority and ethnic minority youth. Assessment of culture-based victimization may well have picked up differences between the ethnic majority and ethnic minority groups. Unfortunately, the sample sizes were too small to assess difference across specific ethnic groups. Similarly, the lack of gender effects were unremarkable given the PECK-SF assesses victimization across a range of forms. Valid subscales that focus on specific forms of victimization may well have identified gender differences (e.g., higher levels of physical victimization in male students; Kyriakides, Kaloyirou, & Lindsay, 2006). Furthermore, multigroup CFA would be a useful procedure to establish whether there is invariance in the measurement properties between groups defined by gender and ethnicity, as our comparison of means assumes such invariance. This detailed procedure, however, was beyond the scope of this early development article.

The PECK Short Form covers a broad range of behaviors including both traditional domains (physical, cultural, relational-verbal) and the more recent cyber victimization. Note that many youth use social media through phone applications and may not categorize these activities as online. To ensure

important information is not missed, future versions of the PECK could include the term “social media.” Despite this limitation, the findings provide evidence to support good internal consistency and structural validity for the full scale. This evidence provides some support for the utility of the PECK-SF in measuring a young person’s victimization experience as a general index of victimization, which includes items that cover the key forms of victimization experienced by young people, including cyber victimization. However, the lack of convergent or discriminant validity evidence limits endorsement of the PECK-SF until this requirement is addressed in future research. The use of a short form measure of victimization may be viewed as pragmatic in research and e-health projects where a smaller number of items are required to ease administration. Overall, the PECK-SF has the potential to provide a rapid assessment of victimization, producing a continuous measure for use as a screen to identify youth who have been bullied.

Declaration of Conflicting Interests

The author(s) declared the following potential conflicts of interest with respect to the research, authorship, and/or publication of this article: Author Hunt is the lead author of the development of the original PECK questionnaire. Authors Prinz, Costa, and Chervonsky declare that they have no conflict of interest.

Funding

The author(s) received no financial support for the research, authorship, and/or publication of this article.

ORCID iD

Caroline Hunt https://orcid.org/0000-0001-7988-1207

References

Austin, S., & Joseph, S. (1996). Assessment of bully/victim problems in 8 to 11 year olds. *British Journal of Educational Psychology*, *66*, 447-456.

Barker, E., Boivin, M., Brendgen, M., Fontaine, N., Arseneault, L., Vitaro, F., . . . Tremblay, R. (2008). Predictive validity and early predictors of peer-victimization trajectories in preschool. *Archives of General Psychiatry*, *65*, 1185-1192.

Berne, S., Frisen, A., Schultze-Krumbholz, A., Scheithauer, H., Naruskov, K., Luik, R., . . . Zukauskiene, R. (2013). Cyberbullying assessment instruments: A systematic review. *Aggression and Violent Behaviour*, *18*, 320-334.

Bond, L., Wolfe, S., Tollit, M., Butler, H., & Patton, G. (2007). A comparison of the Gatehouse Bullying Scale and the peer relations questionnaire for students in secondary school. *Journal of School Health*, *77*, 75-79.

Boulton, M. J., Bucci, E., & Hawker, D. S. J. (1999). Swedish and English secondary school pupils’ attitudes towards, and conceptions of, bullying: Concurrent

links with bully/victim involvement. *Scandinavian Journal of Psychology*, *40*, 277-284.

Callaghan, S., & Joseph, S. (1995). Self-concept and peer victimization among school children. *Personality and Individual Differences*, *18*, 161-163.

Chervonsky, E., & Hunt, C. (2019). Emotion regulation, mental health, and social wellbeing in a young adolescent sample: A concurrent and longitudinal investigation. *Emotion*, *19*, 270-282. doi:10.1037/emo0000432

Coie, J., Dodge, K., & Coppotelli, H. (1982). Dimensions and types of social status: A cross-age perspective. *Developmental Psychology*, *74*, 238-256.

Cook, C. R., Williams, K. R., Guerra, N. G., Kim, T. E., & Sadek, S. (2010). Predictors of bullying and victimization in childhood and adolescence: A meta-analytic investigation. *School Psychology Quarterly*, *25*, 65-83.

Cross, D., Shaw, T., Hearn, L., Epstein, M., Monks, H., Lester, L., & Thomas, L. (2009). *Australian Covert Bullying Prevalence Study (ACBPS)*. Perth, Australia: Child Health Promotion Research Centre, Edith Cowan University.

Demaray, M. K., Malecki, C. K., Secord, S. M., & Lyell, K. M. (2013). Agreement among students,' teachers,' and parents' perceptions of victimization by bullying. *Children and Youth Services Review*, *35*, 2019-2100.

Fekkes, M., Pijpers, F., Fredriks, A., Vogels, T., & Verloove-Vanhorick, S. (2006). Do bullied children get ill, or do ill children get bullied? A prospective cohort study on the relationship between bullying and health-related symptoms. *Journal of Pediatrics*, *117*, 1568-1574.

Felix, E. D., Sharkey, J. D., Green, J. G., Furlong, M. J., & Tanigwa, D. (2011). Getting precise and pragmatic about the assessment of bullying: The development of the California Bullying Victimization Scale. *Aggressive Behavior*, *37*, 234-247.

Hawker, D. S. J., & Boulton, M. J. (2000). Twenty years' research on peer victimization and psychosocial maladjustment: A meta-analytic review of cross-sectional studies. *Journal of Child Psychology and Psychiatry*, *41*, 441-455.

Hu, L., & Bentler, P. M. (1999). Cutoff criteria for fit indexes in covariance structure analysis: Conventional criteria versus new alternatives. *Structural Equation Modeling*, *6*, 1-55.

Hunt, C. (2007). The effect of an education program on attitudes and beliefs about bullying and bullying behaviour in junior secondary school students. *Child and Adolescent Mental Health*, *12*, 21-26.

Hunt, C., Peters, L., & Rapee, R. (2012). Development of a measure of the experience of being bullied in youth. *Psychological Assessment*, *24*, 156-165.

Jensen, C. D., & Steele, R. G. (2010). Validation of the Perceptions of Teasing Scale (POTS) in a preadolescent sample: Associations with attitudes towards physical activity. *Child Health Care*, *39*, 249-265.

Jia, M., & Mikami, A. (2018). Issues in the assessment of bullying: Implications for conceptualizations and future directions. *Aggression and Violent Behavior*, *41*, 108-118.

Klomek, A. B., Marrocco, F., Kleinman, M., Schonfeld, I. S., & Gould, M. S. (2007). Bullying, depression, and suicidality in adolescents. *Journal of the American Academy of Child Adolescent Psychiatry*, *46*, 40-49.

Kowalski, R. M., Giumetti, G. W., Schroeder, A. N., & Lattanner, M. R. (2014). Bullying in the digital age: A critical review and meta-analysis of cyberbullying research among youth. *Psychological Bulletin*, *140*, 1073-1137.

Kyriakides, L., Kaloyirou, C., & Lindsay, G. (2006). An analysis of the Revised Olweus Bully/Victim Questionnaire using the Rasch measurement model. *British Journal of Educational Psychology*, *76*, 781-801.

Lee, T., & Cornell, D. (2009). Concurrent validity of the Olweus Bully/Victim Questionnaire. *Journal of School Violence*, *9*, 56-73.

Marks, P. E. L., Babcock, B., Cillessen, A. H. N., & Crick, N. R. (2013). The effects of participation rate on the internal reliability of peer nomination measures. *Social Development*, *22*, 609-622.

Menesini, E., & Salmivalli, C. (2017). Bullying in schools: The state of knowledge and effective interventions. *Psychology, Health & Medicine*, *22*(Suppl. 1), 240-253.

Muthén, L. K., & Muthén, B. O. (2011). *Mplus User's Guide. 1998–2011*. Los Angeles, California: Muthén and Muthén.

Nguy, L., & Hunt, C. J. (2004). Ethnicity and bullying: A study of Australian high school students. *Educational and Child Psychology*, *21*, 78-94.

Olweus, D. (1996). *The Revised Olweus Bully/Victim Questionnaire*. Mimeo, Norway: Research Centre for Health Promotion (HEMIL Centre), University of Bergen.

Pouwels, J. L., Lansu, T. A. M., & Cillessen, H. N. (2016). Peer victimization in adolescence: Concordance between measures and associations with global and daily internalizing problems. *Journal of Adolescence*, *53*, 195-206.

Reijntjes, A., Kamphuis, J. H., Prinzie, P., & Telch, M. J. (2010). Peer victimization and internalizing problems in children: A meta-analysis of longitudinal studies. *Child Abuse and Neglect*, *34*, 244-252.

Rizopoulos, D. (2006). ltm: An R package for latent variable modeling and item response theory analyses. *Journal of Statistical Software*, *17*(5), 1-25.

Rupp, S., Elliott, S. N., & Gresham, F. M. (2018). Assessing elementary students' bullying and related social behaviors: Cross-informant consistency across school and home environments. *Children and Youth Services Review*, *93*, 458-466.

Samejima, F. (1969). Estimation of latent ability using a response pattern of graded scores. *Psychometrika Monograph Supplement*, *34*, 139-139.

Schäfer, M., Werner, N. E., & Crick, N. R. (2002). A comparison of two approaches to the study of negative peer treatment: General victimization and bully/victim problems among German schoolchildren. *British Journal of Developmental Psychology*, *20*, 281-306.

Scholte, R. J. H., Burk, W. J., & Overbeck, G. (2013). Divergence in self- and peer-reported victimization and its association to concurrent and prospective adjustment. *Journal of Youth and Adolescence*, *42*, 1789-1800.

Smith, P. K., Cowie, H., Olafsson, R. F., & Liefooghe, A. P. D. (2002). Definitions of bullying: A comparison of terms used, and age and gender differences, in a fourteen-country international comparison. *Child Development*, *73*, 1119-1133.

Solberg, M. E., & Olweus, D. (2003). Prevalence estimation of school bullying with the Olweus Bully/Victim Questionnaire. *Aggressive Behavior*, *29*, 239-268.
Vaillancourt, T., Brittain, H., McDougall, P., & Duku, E. (2013). Longitudinal links between childhood peer victimization, internalizing and externalizing problems, and academic functioning: Developmental cascades. *Journal Abnormal Child Psychology*, *41*, 1203-1215.
Vessey, J., Duffy, M., O'Sullivan, P., & Swanson, M. (2003). Assessing teasing in school-age youth. *Issues in Comprehensive Pediatric Nursing*, *26*, 1-11.
World Health Organization. (2002). *World report on violence and health* (E. G. Krug, L. L. Dahlberg, & J. A. Mercy, Eds.). Geneva: World Health Organization. Retrieved from http://whqlibdoc.who.int/publications/2002/9241545615_eng.pdf

Author Biographies

Karoline Prinz has a PhD from the University of Sydney and has a clinical master's degree from the University of Vienna. Her research interests include the treatment of anxiety disorders in children and adolescents, e-mental health, and school-based bullying. She currently works at the Medical University Hospital Vienna, Department of Paediatrics and Adolescent Medicine.

Daniel Costa has a PhD in psychology from the University of Sydney. His research interests are primarily in theoretical and methodological issues pertaining to health questionnaire data, particularly pain, fear of cancer recurrence, and quality of life.

Elizabeth Chervonsky has a doctor of clinical psychology and PhD from the University of Sydney. Her research interests are in emotion regulation, social well-being, and clinical psychology in children, adolescents, and adults. She currently works in clinical practice.

Caroline Hunt is professor in the school of psychology at the University of Sydney. She leads the Clinical Psychology Unit. Her research interests include school-based bullying, and the nature and treatment of anxiety disorders across the lifespan.
