# Supplementary material for: Bullying victimization and suicidal ideation among Chinese adolescents: a moderated mediation model of depressive symptoms and perceived family economic strain
Source: BMC Public Health. 2025 Jan 30;25:393. doi: 10.1186/s12889-025-21579-w (PMC11783786; doi:10.1186/s12889-025-21579-w)
Supplement: Supplementary file 4 — Supplementary Material 4 [file 12889_2025_21579_MOESM4_ESM.docx]

| **Table 1** Moderated mediation analysis of Han ethnicity and minority ethnicity adolescents | | | | | | | | | | | | |
| --- | --- | --- | --- | --- | --- | --- | --- | --- | --- | --- | --- | --- |
| Variables | Han ethnicity | | | | | | Minority ethnicity adolescents | | | | | |
|  | DS | | | SI | | | DS | | | SI | | |
|  | *β* | *t* | 95%*CI* | *β* | *t* | 95%*CI* | *β* | *t* | 95%*CI* | *β* | *t* | 95%*CI* |
| BV | 1.153 *** | 17.421 | (1.023, 1.283) | 0.143** | 2.623 | (0.036,0.251) | 1. 163*** | 15. 668 | (1.018,1.309) | 0.063 | 1.019 | (-0.058, 0.185) |
| PFES | 0 613*** | 4.635 | (0.354, 0.872) | -0.028 | -0.288 | (-0.218, 0.162) | 0.635*** | 4.191 | (0.338, 0.933) | 0.072 | 0.639 | (-0.149, 0.292) |
| DS |  |  |  | 0.559*** | 23.285 | (0.512, 0.606) |  |  |  | 0.586*** | 21.589 | (0.533, 0.639) |
| BV*PFES | -0.013 | -1.883 | (-0.027,0.001) | 0.007 | 1.136 | (-0.005, 0.018) | -0.022** | -2. 769 | (-0.037, -0.006) | 0.003 | 0.503 | (-0.010, 0.016) |
| DS*PFES |  |  |  | -0.003 | -0.932 | (-0.008, 0.003) |  |  |  | -0.004 | -1. 333 | (-0.010, 0.002) |
| Note: 95%*CI*: the 95 %confidence interval. **: *p*< 0.01; ***：*p*< 0.001. Age, Gender, Only child, Caregiver(s) are analyzed as control variables. BV: Bullying victimization; PFES: Perceived family economic strain; DS: Depressive symptoms; SI: Suicidal ideation. | | | | | | | | | | | | |

1. For the Han and ethnic minorities
2. For residential areas

| **Table 2a** Moderated mediation analysis(City) | | | | | | | |
| --- | --- | --- | --- | --- | --- | --- | --- |
| Variable | Model 1（DS） | | | | Model 2（SI） | | |
|  | *β* | *t* | 95%*CI* | | *β* | *t* | 95%*CI* |
| BV | 1.164*** | 30.767 | (1.089,1.238) | | 0.137*** | 4. 423 | (0.135,0.243) |
| PFES | 0.431*** | 8.809 | (0.335,0.527) | | 0.066 | 1.792 | (-0.009,0.112) |
| DS |  |  |  |  | 0.579*** | 47.601 | (0.518, 0.564) |
| BV*PFES | -0.001 | -0.072 | (-0.020,0.018) | | -0.002 | 0.253 | (-0.018, 0.014) |
| DS*PFES |  |  |  |  | -0.003 | -0.732 | (-0.009, 0.004) |
| Note: 95%*CI*:the 95 %confidence interval. ***：*p*< 0.001. Age, Gender, Only child, Caregiver(s) are analyzed as control variables.  BV: Bullying victimization; PFES: Perceived family economic strain; DS: Depressive symptoms; SI: Suicidal ideation. | | | | | | | |

| **Table 2b** Moderated mediation analysis(Town) | | | | | | | |
| --- | --- | --- | --- | --- | --- | --- | --- |
| Variable | Model 1（DS） | | | | Model 2（SI） | | |
|  | *β* | *t* | 95%*CI* | | *β* | *t* | 95%*CI* |
| BV | 1.100*** | 16. 351 | (0.968, 1.232) | | 0.233*** | 4.189 | (0.124,0.342) |
| PFES | 0.404*** | 4.823 | (0.240, 0.569 | | 0.079 | 1.272 | (--0. 043,0.201) |
| DS |  |  |  |  | 0.531*** | 22.789 | (0.485, 0.577) |
| BV*PFES | -0.021 | -1. 411 | (-0.051,0.008) | | 0.004 | 0. 307 | (-0.023, 0.032) |
| DS*PFES |  |  |  |  | 0.004 | 0.643 | (-0.009, 0.018) |
| Note: 95%*CI*:the 95 %confidence interval. ***：*p*< 0.001. Age, Gender, Only child, Caregiver(s) are analyzed as control variables.  BV: Bullying victimization; PFES: Perceived family economic strain; DS: Depressive symptoms; SI: Suicidal ideation. | | | | | | | |

| **Table 2c** Moderated mediation analysis(Rural) | | | | | | | |
| --- | --- | --- | --- | --- | --- | --- | --- |
| Variable | Model 1（DS） | | | | Model 2（SI） | | |
|  | *β* | *t* | 95%*CI* | | *β* | *t* | 95%*CI* |
| BV | 0.887*** | 25.866 | (0.820, 0.955) | | 0.118*** | 4.175 | 0.063,0.173) |
| PFES | 0.261*** | 6.268 | (0.179, 0.342） | | 0.054 | 1.740 | (-0. 007,0.116) |
| DS |  |  |  |  | 0.507*** | 37.578 | (0.480, 0.533) |
| BV*PFES | -0.010 | -1. 373 | (-0.024,0.004) | | 0.009 | 1.545 | (-0.003, 0.021) |
| DS*PFES |  |  |  |  | -0.001 | -0.169 | (-0.007, 0.006) |
| Note: 95%*CI*:the 95 %confidence interval. ***：*p*< 0.001. Age, Gender, Only child, Caregiver(s) are analyzed as control variables.  BV: Bullying victimization; PFES: Perceived family economic strain; DS: Depressive symptoms; SI: Suicidal ideation. | | | | | | | |

| **Table 3** The moderating effect of high and low perceived family economic stress levels on bullying victimization and depressive symptoms | | | | |
| --- | --- | --- | --- | --- |
| Perceived family economic strain | Effect | *t* | 95%*CI* |  |
| 4 | 1.076 | 22.392 | (0.982, 1.17) | |
| 7.347 | 1.003 | 29.426 | (0.936, 1.069) | |
| 10.83 | 0.926 | 24.326 | (0.852, 1.001) | |
| Note: 95%*CI*:the 95 %confidence interval | | | | |
